# Supplementary material for: Improving classification based on physical surface tension-neural net for the prediction of psychosocial-risk level in public school teachers
Source: PeerJ Comput Sci. 2021 May 26;7:e511. doi: 10.7717/peerj-cs.511 (PMC8176537; doi:10.7717/peerj-cs.511)
Supplement: Supplemental Information 1 [file peerj-cs-07-511-s001.doc]

|  | **CONSENTIMIENTO INFORMADO DE LA APLICACIÓN DE LA BATERIA DE RIESGO PSICOSOCIAL**  PROYECTO DE INVESTIGACIÓN | FECHA DE DILIGENCIAMIENTO |
| --- | --- | --- |
|  |  | AAAA - MM – DD |
|  |  |  |

| Yo, ______________________________________________________ servidor público identificado(a) con cédula de ciudadanía No. ______________________ de ____________________, declaro haber sido plenamente informado (a) sobre el proceso de evaluación de los Factores de Riesgo Psicosocial que adelantará el **COLEGIO XXXXXXXXXX,** en cumplimiento de la Resolución 2646 de 2008, el cual consta de lo siguiente:   1. La evaluación tiene como objetivo la identificación y valoración de los factores psicosociales y sus posibles efectos sobre mi salud y/o desempeño, con el fin de establecer Planes de Intervención que respondan a las características y necesidades detectadas en los servidores públicos de la entidad. 2. La metodología de evaluación consiste en la aplicación de 1 instrumento de medición de los factores de riesgo psicosocial, debidamente validados para la población trabajadora colombiana, dichos instrumentos son:  - Ficha de datos generales - Cuestionario de factores de riesgo psicosocial intralaboral  1. Los resultados obtenidos serán de absoluta RESERVA. Y solo se utilizarán para fines científicos de la investigación. 2. La información individual utilizada para la presente evaluación será manejada con absoluta confidencialidad y será usada exclusivamente para los fines inherentes a la Salud Ocupacional. La misma, será revelada únicamente con mi consentimiento o el de mi representante legal, excepto en aquellas circunstancias particulares en que no hacerlo represente un evidente daño para mí o para otros. 3. Mi participación en esta evaluación es estrictamente voluntaria; es decir que yo puedo libremente decidir participar en el proceso o rehusarme a hacerlo, sin que esta decisión represente ningún perjuicio para mi persona. 4. Me han informado que puedo formular las preguntas sobre el proceso de evaluación, en cualquier momento del mismo, al profesional que acompaña el proceso (Psicólogo con posgrado en Salud Ocupacional y licencia vigente). 5. En el evento en que el profesional a cargo lo requiera, podrá contactarme por vía telefónica o correo electrónico.   Con base en la información que me han suministrado sobre el proceso de evaluación de los factores psicosociales, declaro libremente que he leído y entendido completamente lo referente al mismo (objetivos, metodología, tratamiento de la información, informes de resultados, participación voluntaria). De igual manera declaro que conozco mi derecho a formular las preguntas que me surjan antes, durante y/o después del proceso de evaluación, con el fin de aclarar lo pertinente.  Teniendo en cuenta lo anterior, expreso libremente mi voluntad de:  *(Marque una X en el recuadro que corresponda, según su decisión y a continuación registre sus datos)*      **SI** Participar en el proceso de evaluación **NO** Participar en el proceso de evaluación  de factores de Riesgo Psicosocial de factores de Riesgo Psicosocial    Nombre del Servidor Público: ________________________________    Documento de Identificación: ________________________________ ____________________________________    FIRMA  Correo electrónico: ________________________________________ |
| --- |
