# Supplementary figures and images for: Improving classification based on physical surface tension-neural net for the prediction of psychosocial-risk level in public school teachers
Source: PeerJ Comput Sci. 2021 May 26;7:e511. doi: 10.7717/peerj-cs.511 (PMC8176537; doi:10.7717/peerj-cs.511)

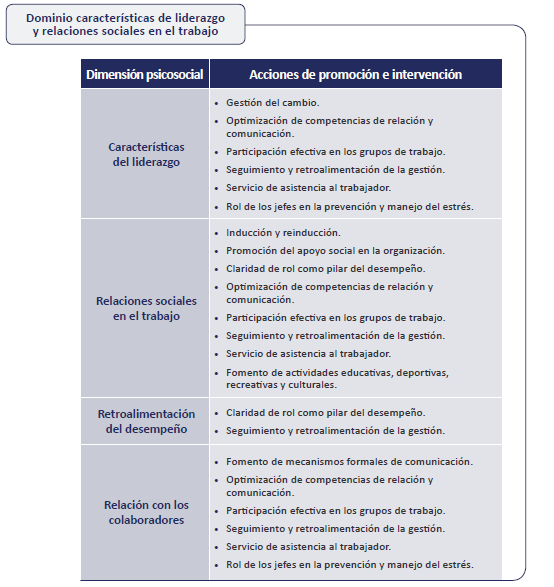

Supplement: Supplemental Information 2 [file peerj-cs-07-511-s002.zip › graf/1.Liderazgo Rel_Soc_Trabj.png]

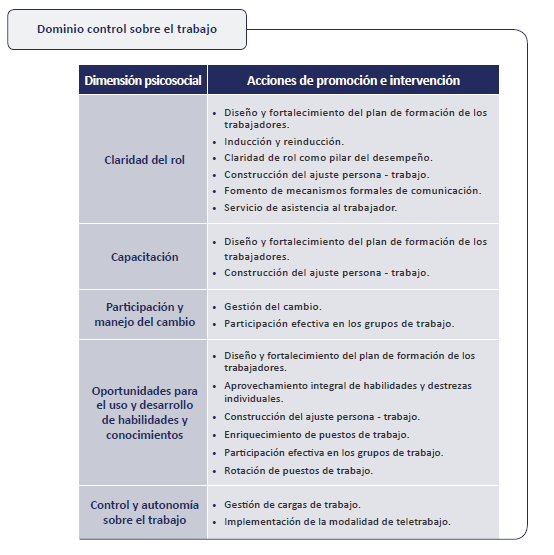

Supplement: Supplemental Information 2 [file peerj-cs-07-511-s002.zip › graf/2.Control.png]

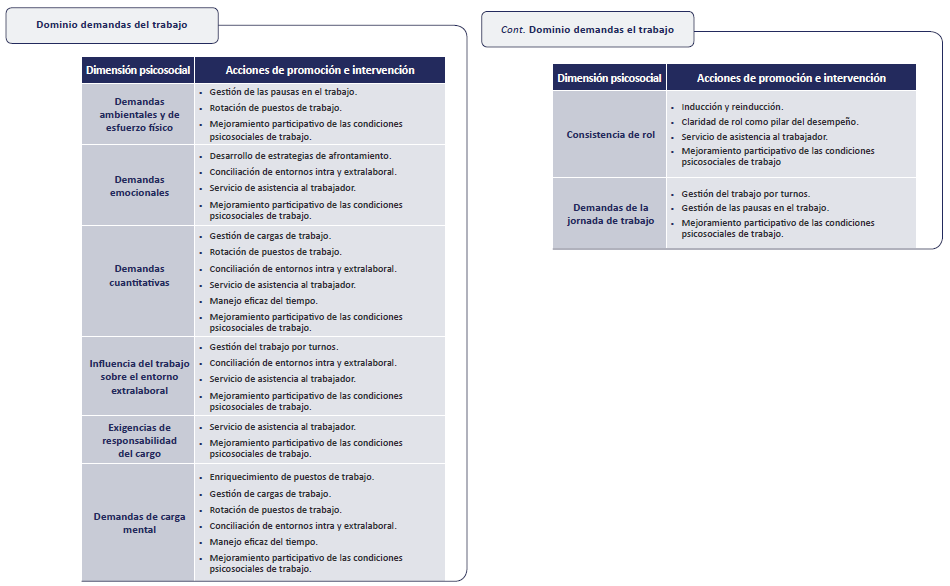

Supplement: Supplemental Information 2 [file peerj-cs-07-511-s002.zip › graf/3.Demandas del trabajo.png]

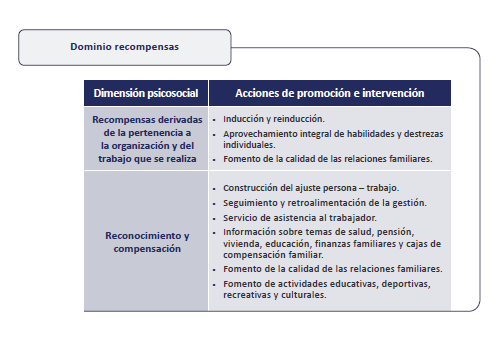

Supplement: Supplemental Information 2 [file peerj-cs-07-511-s002.zip › graf/4.Recompensas.png]

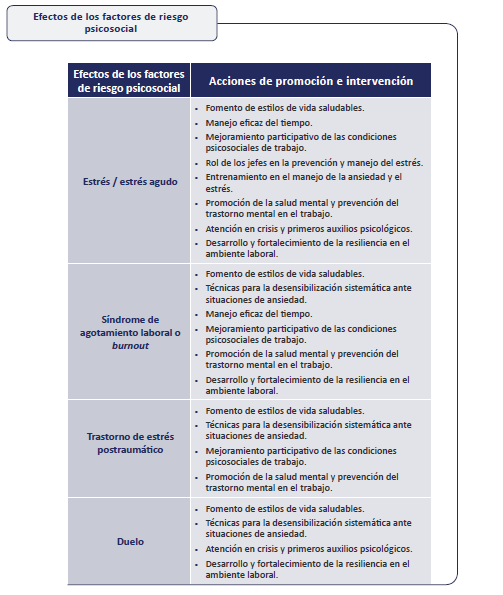

Supplement: Supplemental Information 2 [file peerj-cs-07-511-s002.zip › graf/5.Efectos de los frp.png]

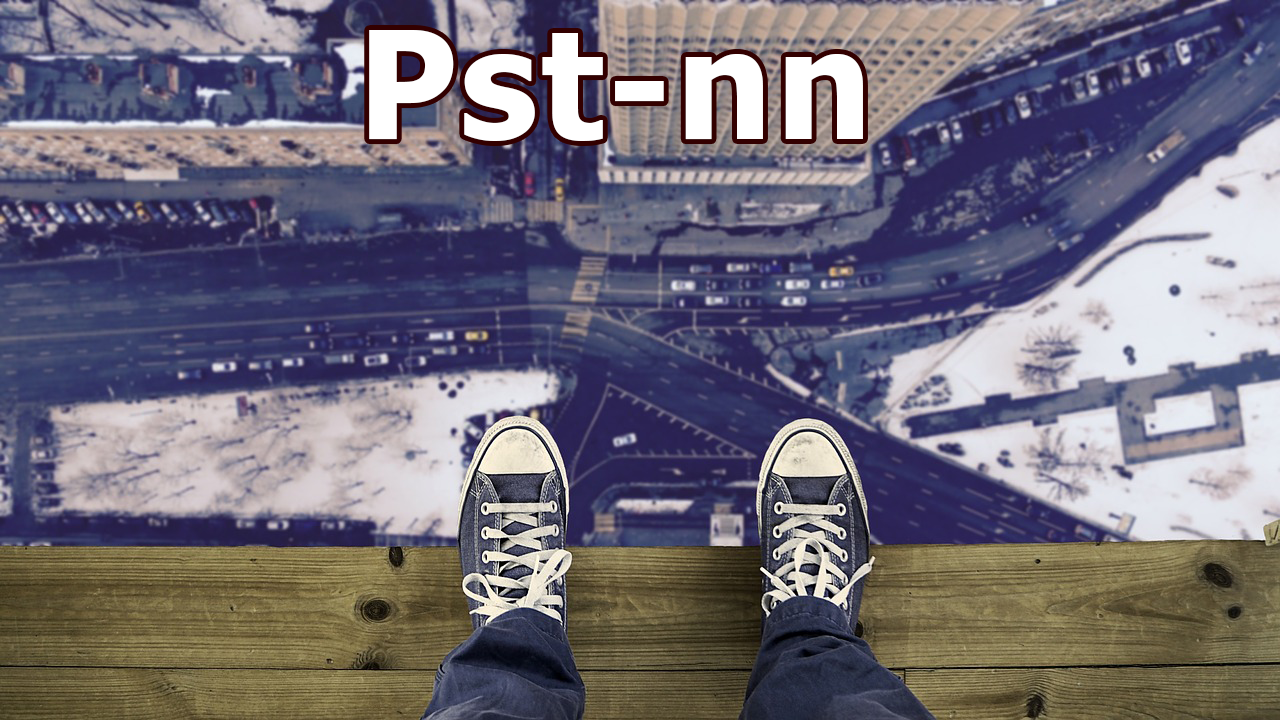

Supplement: Supplemental Information 2 [file peerj-cs-07-511-s002.zip › graf/fondo.png]
